# Glucocorticoid receptor signaling in ventral tegmental area neurons increases the rewarding value of a high-fat diet in mice

**DOI:** 10.1038/s41598-021-92386-7

**Published:** 2021-06-18

**Authors:** Akira Mizoguchi, Ryoichi Banno, Runan Sun, Hiroshi Yaginuma, Keigo Taki, Tomoko Kobayashi, Mariko Sugiyama, Taku Tsunekawa, Takeshi Onoue, Hiroshi Takagi, Daisuke Hagiwara, Yoshihiro Ito, Shintaro Iwama, Hidetaka Suga, Taku Nagai, Kiyofumi Yamada, Hiroshi Arima

**Affiliations:** 1 Department of Endocrinology and Diabetes, Nagoya University Graduate School of Medicine, 65 Turumai-cho, Showa-ku, Nagoya, Aichi 466-8560 Japan; 2 Physical Fitness and Sports, Research Center of Health, Nagoya University, Furo-cho, Chikusa-ku, Nagoya, Aichi 464-0814 Japan; 3 Department of CKD Initiatives/Nephrology, Nagoya University Graduate School of Medicine, 65 Turumai-cho, Showa-ku, Nagoya, Aichi 466-8560 Japan; 4 Department of Neuropsychopharmacology and Hospital Pharmacy, Nagoya University Graduate School of Medicine, 65 Turumai-cho, Showa-ku, Nagoya, Aichi 466-8560 Japan; 5 Division of Behavioral Neuropharmacology, Project Office for Neuropsychological Research Center, Fujita Health University, 1-98 Dengakugakubo, Kutsukake-cho, Toyoake, Aichi 470-1192 Japan

**Keywords:** Feeding behaviour, Endocrine system and metabolic diseases, Reward

## Abstract

The reward system, which consists of dopaminergic neurons projecting from the ventral tegmental area (VTA) to the nucleus accumbens and caudate-putamen in the striatum, has an important role in the pathogenesis of not only drug addiction but also diet-induced obesity. In the present study, we examined whether signaling through glucocorticoid receptors (GRs) in the reward system affects the rewarding value of a high-fat diet (HFD). To do so, we generated mice that lack functional GRs specifically in dopaminergic neurons (D-KO mice) or corticostriatal neurons (CS-KO mice), subjected the mice to caloric restriction stress conditions, and evaluated the rewarding value of a HFD by conditioned place preference (CPP) test. Caloric restriction induced increases in serum corticosterone to similar levels in all genotypes. While CS-KO as well as WT mice exhibited a significant preference for HFD in the CPP test, D-KO mice exhibited no such preference. There were no differences between WT and D-KO mice in consumption of HFD after fasting or cognitive function evaluated by a novel object recognition test. These data suggest that glucocorticoid signaling in the VTA increases the rewarding value of a HFD under restricted caloric stress.

## Introduction

Feeding behavior is known to be regulated by not only the hypothalamus but also the reward system^1,2^, in which dopaminergic neurons in the ventral tegmental area (VTA) and substantia nigra project to the nucleus accumbens (NAc) and caudate-putamen (CPu), respectively, in the striatum^3^. This neurocircuit is activated by consuming palatable food, processes information related to food reward, and provides emotional satiety^4^. Studies have also revealed that the reward system is involved in the pathophysiology of diet-induced obesity^3,5,6^, which is a major health issue in many countries^7^.

A high-fat diet (HFD), which consists of a highly palatable food, is suggested to have addiction-like properties such as cocaine or heroin^8^. There are several lines of evidence to support that rodents become more addicted to narcotic substances under stress conditions or after administration of glucocorticoids^9,10^. Stress also promotes consumption of palatable food^11^, and previous studies have shown that increases in serum cortisol concentrations in response to stress are closely correlated with the amount of snack intake in human subjects^12^, and that mice which were moderately food-restricted in advance showed a significant increase in binge eating of a palatable high-fat food during stress exposure^13^. These results suggest that glucocorticoid signaling activated by stress can stimulate the reward system and thereby increase intake of palatable food, although the detailed mechanisms have not yet been clarified.

In the present study, we generated mice that lack functional GRs specifically in dopaminergic and corticostriatal neurons, and examined whether signaling through GRs in the reward system affects the rewarding value of a HFD by using a conditioned place preference (CPP) test, in order to investigate the role of glucocorticoid signaling in the reward system.

## Results

### Generation of dopaminergic neuron-specific and corticostriatal neuron-specific GR-deficient mice

**Figure 1:**
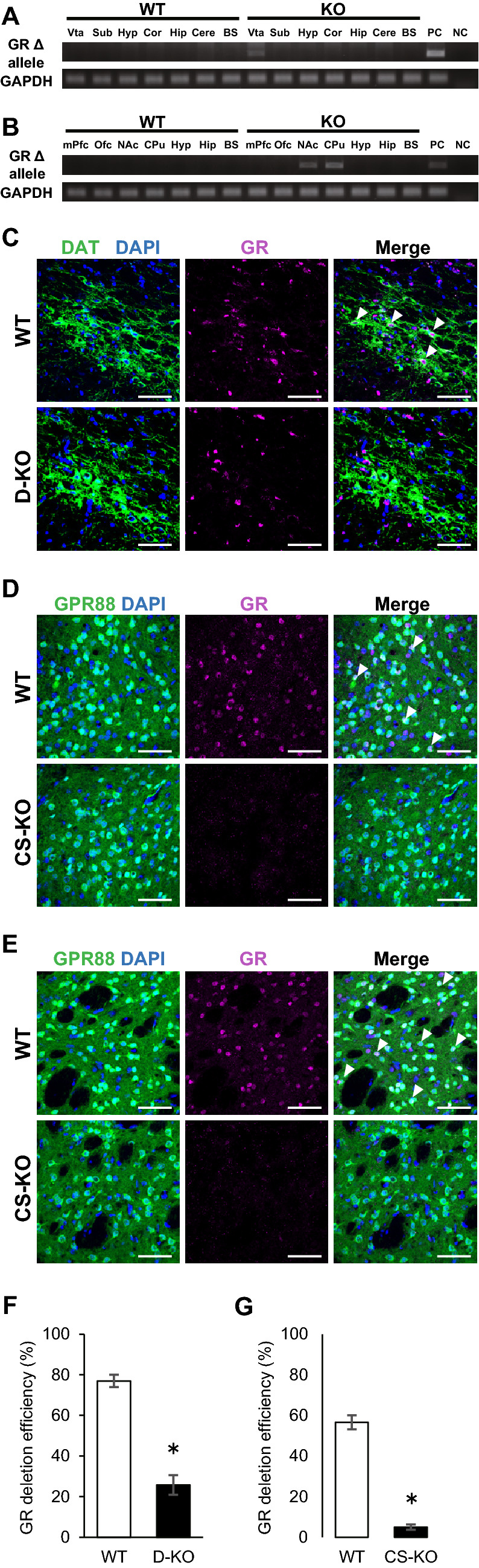
Generation of dopaminergic neuron-specific and corticostriatal neuron-specific GR deficient mice. (**A**,**B**) Detection of deletion of *GR* alleles (Δ) in *DAT-GR*^-/-^ (D-KO) mice (**A**) and *CPR88-GR*^-/-^ (CS-KO) mice (**B**) compared with *GR*^+/+^ mice (WT). Vta, ventral tegmental area; Sub, substantia nigra; Hyp, hypothalamus; Cor, cerebral cortex; Hip, hippocampus; Cer, cerebellum; BS, brainstem; mPfc, medial prefrontal cortex; Ofc, orbitofrontal cortex; NAc, nucleus accumbens; CPu, caudate-putamen; PC, positive control; NC, negative control. PCR reaction with *GAPDH* was used as an internal control. Deletion of the GR allele in D-KO mice was only detected in DNA extracts from the VTA (**A**). Similarly, deletions of the GR allele in CS-KO mice were detected in DNA extracts from the NAc and CPu (**B**). (**C**) Immunostaining of DAT and glucocorticoid receptor (GR) in the VTA of mice. Representative photographs show the staining of DAT (green), GR (red) and DAPI (blue) in the VTA in WT and D-KO mice. (**D**,**E**) Immunostaining of GR in the NAc (**D**) and CPu (**E**) of mice. Representative photographs show the staining of GR (red) and DAPI (blue) in NAc and CPu in *GPR88-GR*^+/+^ *R26GRR* mice (WT) and *GPR88-GR*^-/-^ *R26GRR* mice (CS-KO). Scale bar: 50 µm. (**F**,**G**) The ratio of GR expressing cell number in the DAT expressing neurons in VTA (**F**) or GPR88 expressing neurons in NAc (**G**) (n = 4 for each group). All values are mean ± SEM. Statistical analyses were performed using unpaired t test. Asterisks denote significant group differences at *p* < 0.05.

To generate dopaminergic neuron-specific GR deficient mice, *GR*^*loxP/loxP*^ mice were crossed with dopamine transporter (DAT)-*Cre* (DAT-Cre) mice. We next crossed *GR*^+*/loxP*^ DAT- Cre mice with *GR*^*loxP/loxP*^ or *GR*^+*/loxP*^ mice to yield *GR*^*loxP/loxP*^ DAT-Cre (hereafter termed D-KO) mice and *GR*^*loxP/loxP*^ littermate controls (hereafter termed WT) mice. GPR88 is an orphan G-protein-coupled receptor that is highly expressed in striatal and cortical neurons^14,15^. To generate corticostriatal neuron-specific GR-deficient mice, *GR*^*loxP/loxP*^ mice were crossed with GPR88-Cre mice. We next crossed *GR*^+*/loxP*^ GPR88-Cre mice with *GR*^*loxP/loxP*^ or *GR*^+*/loxP*^ mice to yield *GR*^*loxP/loxP*^ GPR88-Cre (hereafter termed CS-KO) mice and *GR*^*loxP/loxP*^ littermate controls (hereafter termed WT) mice. Deletion of the GR allele (Δ) in D-KO mice was only detected in DNA extracts from the VTA (Fig. 1A). Similarly, deletions of the GR allele (Δ) in CS-KO mice were detected in DNA extracts from the NAc and CPu (Fig. 1B). In contrast, no deletion of the floxed alleles was detected by PCR in WT mice (Fig. 1A,B). Immunostaining for both DAT and GR revealed that GRs were expressed in dopaminergic neurons of the VTA in WT mice, whereas GR-expressing cells in the VTA were rarely detected in D-KO mice (Fig. 1C). To assess the localization of GPR88 and GR, we crossed CS-KO mice to ROSA26 Cre-reporter knock-in mice (hereafter termed R26GRR), in which green fluorescence changed to red fluorescence in Cre-recombined cells^16^. *GR*^*loxP/loxP*^* GPR88-Cre R26GRR* mice expressed tdsRed-positive cells in the NAc and CPu, and GR immunostaining revealed that GR was expressed in the tdsRed-positive cells in the NAc and CPu in WT but not in CS-KO mice (Fig. 1D,E), while GR was expressed in the tdsRed-positive cells in mPfc and Ofc in WT as well as CS-KO mice (Supplementary Figure 1). The ratio of GR expressing cell number in the dopaminergic neurons in VTA or GPR88 expressing neurons in NAc was significantly decreased in D-KO mice (about 30%) or CS-KO (about 10%) mice compared with WT (Fig. 1F,G).

### Glucocorticoid signaling in the VTA increases the rewarding value of a HFD under caloric restriction stress

**Figure 2:**
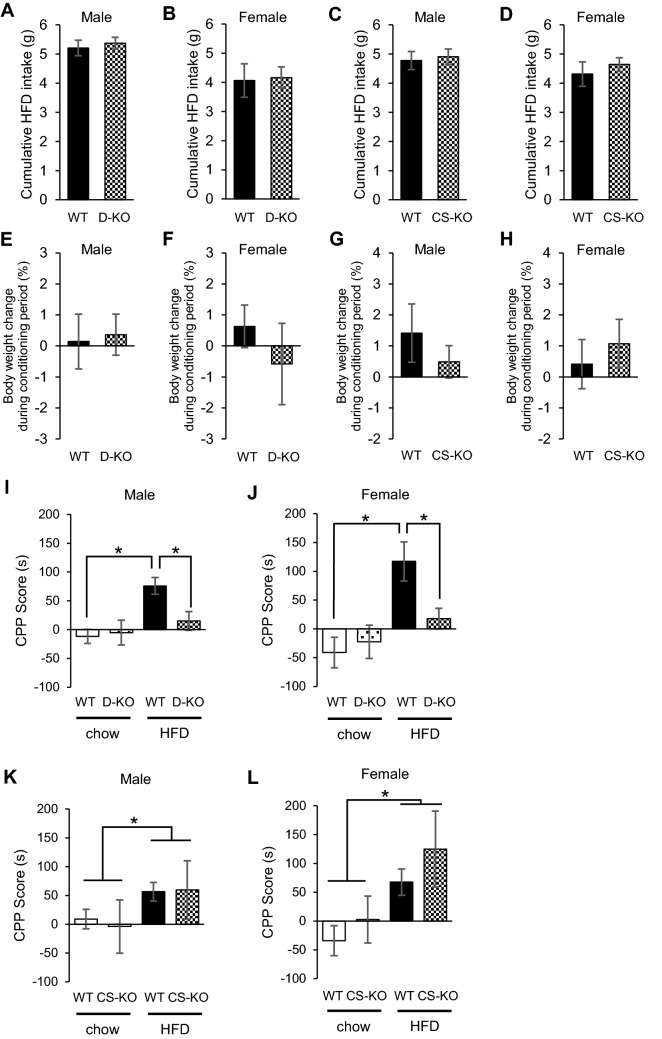
Glucocorticoid signaling in VTA increases the rewarding value of a HFD under caloric restriction stress. (**A–D**) The amount of HFD consumed during CPP conditioning session. There were no differences between WT and D-KO (**A: male** n = 9–13, **B: female** n = 7–10 for each group), or CS-KO (**C: male** n = 8–14, **D: female** n = 8–14 for each group). (**E–H**) Body weight change during the CPP conditioning session. There were no differences between WT and D-KO (**E: male** n = 9–13, **F: female** n = 7–10 for each group), or CS-KO mice (**G: male** n = 8–14, **H: female** n = 8–14 for each group). (**I–L**) Results of the CPP test under caloric restriction stress. WT mice exhibited a significant preference for the HFD-paired compartment, while D-KO mice exhibited no preference for the HFD-paired compartment, in both males and females (**I: male** n = 9–13, **J: female** n = 7–10 for each group). Both WT and CS-KO mice exhibited a significant preference for the HFD-paired compartment, with no difference between genotypes (**K: male** n = 8–14, **L: female** n = 8–14 for each group). All values are mean ± SEM. Statistical analyses were performed using unpaired t test (**A–H**) or two-way factorial ANOVA (**I–L**) followed by Bonferroni post hoc test. Asterisks denote significant group differences at *p* < 0.05 for (**I–L**).

To investigate the role of glucocorticoid signaling in VTA, we performed experiments under stressed conditions in which corticosterone, a glucocorticoid hormone in mice, is expected to increase. We applied the CPP test, with which rewarding value of a palatable food can be evaluated^17–19^. Mice were placed on the protocol for caloric restriction (access to food only for 4 h during the light cycle) for 6 days prior to CPP preconditioning, and their BWs were reduced to 85%-90% of that at the start of the experiment. Caloric restriction was maintained throughout the session, so that mice underwent the experiment under a stressed condition. Both BW and cumulative intake of HFD were similar among WT, D-KO, and CS-KO mice under caloric restriction during the conditioning session (Fig. 2A–H). While WT and CS-KO mice exhibited a significant preference for the HFD [Male, effect of diet: *F*(1,39) = 3.935, p < 0.05; effect of genotype: *F*(1,39) = 0.008, p = 0.931; effect of interaction between diet and genotype: *F*(1,39) = 0.115, p = 0.737; Female, effect of diet: *F*(1,43) = 12.708, p < 0.01; effect of genotype: *F*(1,43) = 2.476, p = 0.123; effect of interaction between diet and genotype: *F*(1,43) = 0.287, p = 0.595 by two-way ANOVA followed by post hoc testing], D-KO mice exhibited no such preference [Male, effect of diet: *F*(1,43) = 12.014, p < 0.01; effect of genotype: *F*(1,43) = 3.292, p = 0.077; effect of interaction between diet and genotype: *F*(1,43) = 3,872, p < 0.05; Female, effect of diet: *F*(1,29) = 15.826, p < 0.01; effect of genotype: *F*(1,29) = 0.347, p = 0.561; effect of interaction between diet and genotype: *F*(1,29) = 7.119, p < 0.05 by two-way ANOVA followed by post hoc testing] (Fig. 2I–L). Conversely, preference for HFD was not observed in the CPP test between WT and D-KO mice under non-stressed condition (Supplementary Figure 2).

### Glucocorticoid signaling in the VTA affects the mRNA expressions associated with dopamine signaling under caloric restriction stress

**Figure 3:**
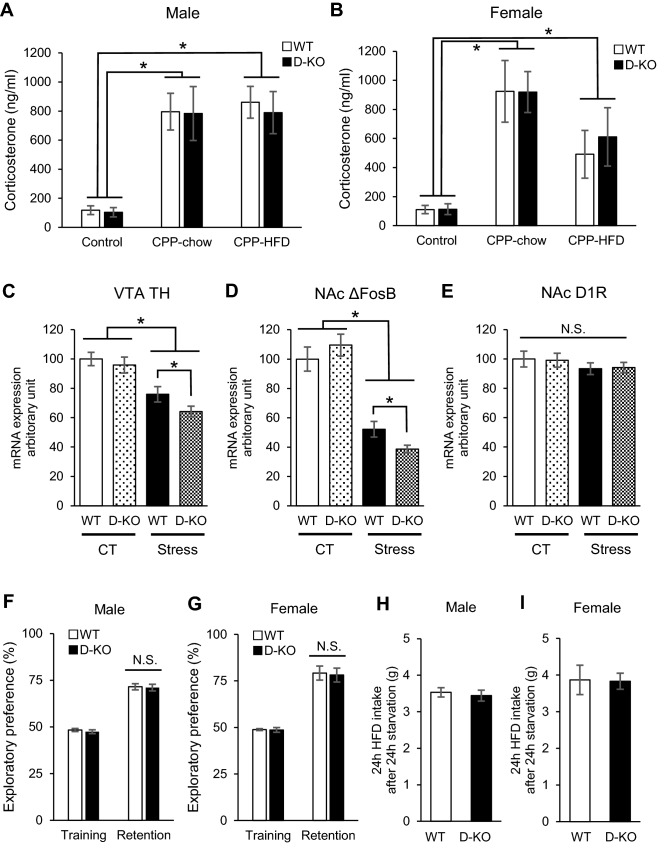
Caloric restriction affected the mRNA expressions of *TH* in VTA and *ΔFosB* in NAc via GR signaling in dopaminergic neurons, while corticosterone levels, cognitive function, and refeeding induced by caloric restriction stress showed no differences. (**A**,**B**) Serum corticosterone levels at the end of the CPP session, compared to control (under no stress). Both D-KO and WT exhibited significantly higher levels of serum corticosterone, with no difference between genotypes nor between feeding conditions (**A: male**, **B: female**, n = 5–7 for each group). (**C–E**) mRNA expressions of *TH* in VTA (**C**), *ΔFosB* (**D**) and *D1R* (**E**) in NAc after caloric restriction (termed ‘Stress’). In the control group, there were no significant differences in the mRNA expression levels between genotypes (**C–E**). The caloric restriction reduced the mRNA expressions of *TH* and *ΔFosB* in both D-KO and WT mice compared to control groups, with significant reduction in D-KO compared to WT (**C**,**D**) (female mice, n = 7–10 for each group). (**F**,**G**) NORT results. There were no significant differences in exploratory preference between D-KO and WT in both the training and retention sessions (**F: male**, **G: female**, n = 9–13 for each group). (**H**,**I**) HFD refeeding after fasting for 24 h. Both D-KO and WT, male (**H**) and female (**I**) mice exhibited no significant differences in the amount of HFD consumed (n = 5–7 for each group). All values are mean ± SEM. Statistical analyses were performed using two-way factorial ANOVA followed by Bonferroni post hoc test (**A-G**) or unpaired t test (**H**, **I**). Asterisks denote significant group differences at *p* < 0.05 vs control for (**A-D**).

Serum levels of corticosterone were significantly increased in all groups subjected to the CPP test for 19 days compared with control mice, and there were no differences in serum corticosterone levels between genotypes [Male, effect of condition: *F*(2,36) = 22.829, p < 0.01; effect of genotype: *F*(1,36) = 0.113, p = 0.738; effect of interaction between condition and genotype: *F*(2,36) = 0.039, p = 0.962; Female, effect of condition: *F*(2,26) = 12.946, p < 0.01; effect of genotype: *F*(1,26) = 0.275, p = 0.604; effect of interaction between condition and genotype: *F*(2,26) = 0.083, p = 0.921 by two-way ANOVA followed by post hoc testing] (Fig. 3A,B). To investigate caloric restriction stress affects the mRNA expression levels associated with dopamine signaling in the mesolimbic system, we subjected female D-KO and WT mice into caloric restriction in which the mice could access to chow for 4 h per day during 19 days, and evaluated the changes of the mRNA expression levels of *tyrosine hydroxylase (TH)* in VTA, *FBJ osteosarcoma oncogene B* (*ΔFosB)* and *dopamine1 receptor D1 (D1R)* in NAc. In the control group that had no caloric restriction, there were no significant differences in the mRNA expression levels of *TH*, *D1R* and *ΔFosB* between genotypes (Fig. 3C–E). The caloric restriction reduced the mRNA expressions of *TH* and *ΔFosB* in both D-KO and WT mice compared to control groups (Fig. 3C,D), whereas there were no differences in *D1R* (Fig. 3E) [*TH*, effect of condition: *F*(1,28) = 38.775, p < 0.01; effect of genotype: *F*(1,28) = 3.516, p = 0.071; effect of interaction between diet and genotype: *F*(1,28) = 0.950, p = 0.338; *ΔFosB*, effect of condition: *F*(1,25) = 104.738, p < 0.01; effect of genotype: *F*(1,25) = 0.305, p = 0.586; effect of interaction between diet and genotype: *F*(1,25) = 4.934, p < 0.05 by two-way ANOVA followed by post hoc testing]. Of note, the mRNA expression levels of *TH* and *ΔFosB* in D-KO were significantly decreased compared to WT under caloric restriction (Fig. 3C,D). NORT was performed to evaluate the cognitive functions of mice. During the training session, both D-KO and WT mice spent equal amounts of time exploring either of the 2 objects and, thus, there was no biased exploratory preference in either group of animals (Fig. 3F,G). Total time spent in the exploration of a new object during the retention session did not differ between D-KO and WT mice (Fig. 3F,G). There were no significant differences in consumption of HFD after 24-h fasting between D-KO and WT mice (Fig. 3H,I). Similar results were obtained after fasting for 12 h or 48 h (data not shown).

### Glucocorticoid signaling in dopaminergic and corticostriatal neurons does not affect energy metabolism

**Figure 4:**
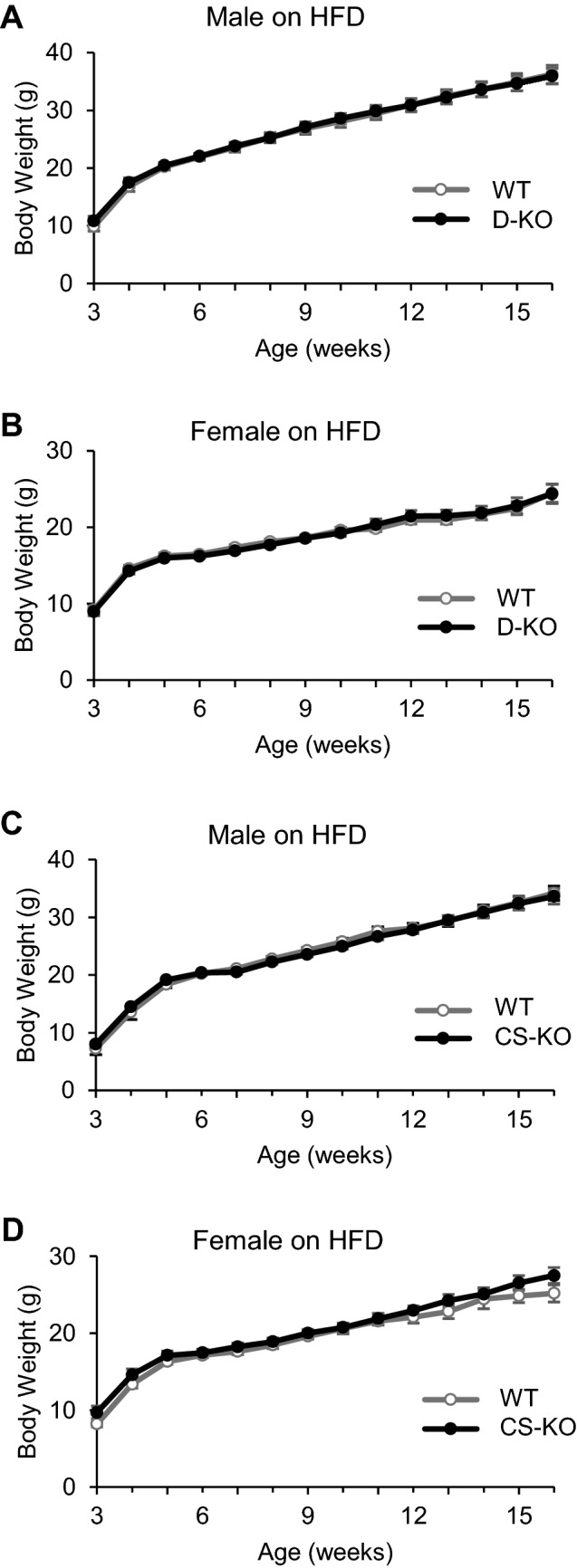
Glucocorticoid signaling in dopaminergic or corticostriatal neurons does not affect energy metabolism. (**A**,**B**) There was no significance between D-KO and WT mice for either male or female (n = 7–12 for each group). (**C**,**D**) There was no significance in body weight between CS-KO and WT mice for either male or female (n = 7–11 for each group). All values are mean ± SEM. Statistical analyses were performed using two-way ANOVA assessed for repeated measures.

**Figure 5:**
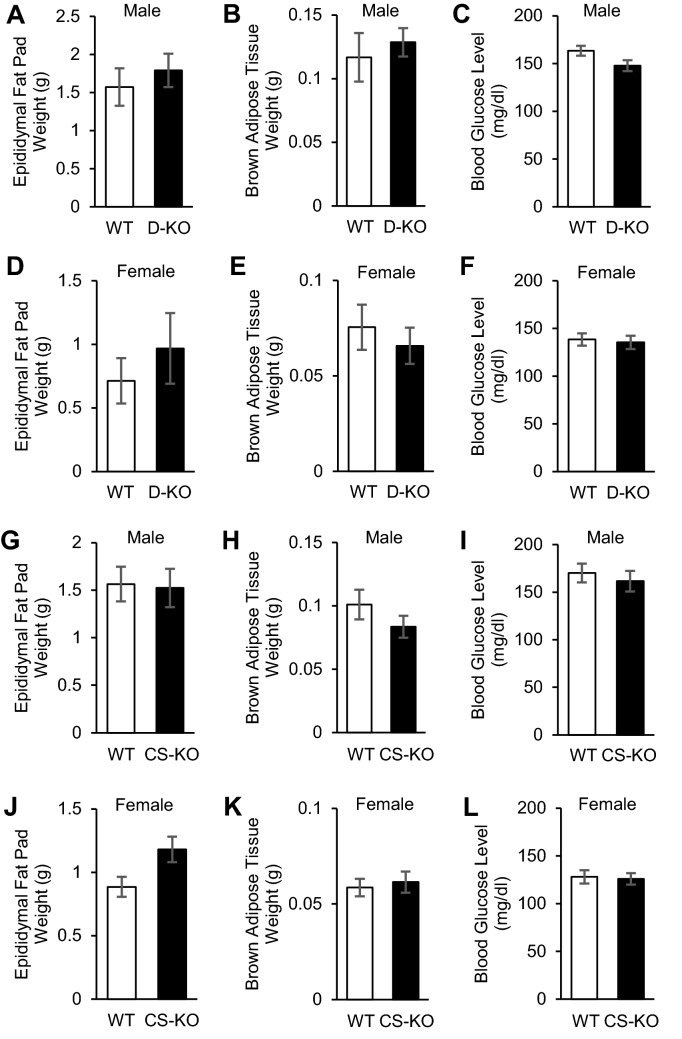
Body components and glucose metabolism were not affected by glucocorticoid receptor signaling in either dopaminergic neurons or corticostriatal neurons. (**A–F**) Epidydimal fat pad weights (**A**,**D**), brown adipose tissue weights (**B**,**E**), and blood glucose levels (**C**,**F**) of D-KO and WT mice at the age of 16 weeks (**A–C**, males, n = 7–9 for each: **D–F**, females, n = 7–12 for each). No significant differences in those parameters were found in either male or female mice. (**G–L**) Epidydimal fat pad weights (**G**,**J**), brown adipose tissue weights (**H**,**K**), and blood glucose levels (**I**,**L**) of CS-KO and WT mice at the age of 16 weeks (**G-I**, males, n = 9–11 for each: (**J–L**) females, n = 7–8 for each). No significant differences were found in those parameters in either male or female mice. All values are mean ± SEM. Statistical analyses were performed using unpaired t test.

Finally, we examined the effects of glucocorticoid signaling in dopaminergic and corticostriatal neurons on energy metabolism. There were no significant differences in BW between WT, D-KO, and CS-KO mice with ad libitum access to HFD (Fig. 4A–D). There were no significant differences in epidydimal fat pad weight, brown adipose tissue weight, and blood glucose levels at the age of 16 weeks between WT, D-KO, and CS-KO mice fed HFD (Fig. 5) or fed chow (data not shown).

## Discussion

In the present study, a significant HFD preference in the CPP test was exhibited by WT and CS-KO mice but not by D-KO mice. Caloric restriction increased serum corticosterone levels similarly in WT and D-KO mice, and both cognitive function and the amount of HFD consumption after fasting were similar between genotypes. Taken together, it is suggested that glucocorticoid signaling in the VTA increases the rewarding value of a HFD under caloric restriction stress.

The CPP test employed in the present study is widely used to assess the rewarding value of a palatable food in murine models^17–19^. Although deletion of GRs in dopaminergic (D-KO mice) or corticostriatal (CS-KO mice) neurons had no effects on energy balance, D-KO but not CS-KO mice showed decreased preference for HFD compared with WT mice in the CPP test. Our data also showed that there was no difference in cognitive function between D-KO and WT mice, suggesting that the difference in CPP score between genotypes was not due to impairment of the acquisition or maintenance of memory. In addition, there were no significant differences in HFD consumption between D-KO and WT mice, suggesting that the motivation for eating was similar between genotypes. Taken together, it appears GR signaling in dopaminergic neurons potentiates the rewarding value of a palatable food.

HFD is generally considered to be a highly palatable food and is preferentially overconsumed^20^. HFD can stimulate the brain reward system independently of its caloric value, and the motivation to consume this palatable food can be high even in the absence of homeostatic energy requirements^2^. It is also reported that food-evoked dopamine release was amplified in food-deprived animals^21^, and that lowering glucose concentration increased glutamatergic miniature excitatory postsynaptic currents onto VTA dopamine neurons^22^. In addition, glucocorticoids are reported to modify postsynaptic dopaminergic transmission in the dorsolateral striatum^23^, and the gene for tyrosine hydroxylase (TH), the rate-limiting enzyme in dopamine biosynthesis, reportedly possesses a transcriptional-stimulatory glucocorticoid response element (GRE)-like sequence (GGCACAGTGTGGTCT) located in the 5’ flanking DNA between positions -2435 and -2421 from the transcription start site^24^. Furthermore, in the present study we found that the mRNA expression levels of *TH* in VTA and *ΔFosB* in NAc in D-KO mice were significantly decreased compared to WT mice under caloric restriction without changing the mRNA expression of *D1R* in NAc, suggesting that the activity of dopaminergic neurons was attenuated due to the lack of GR in VTA. Thus, it is possible that increased corticosterone under calorie-restricted conditions enhances the activity of dopaminergic neurons in the VTA through the GRE, although further study is necessary to prove this. Conversely, our data showed that the deletion of the GR in corticostriatal neurons did not affect the rewarding value of HFD. Consistent with our findings, a previous study reported that GRs in dopaminoceptive neurons did not affect the score on the CPP test, in which the preference for chocolate with cereals was measured under caloric restriction by using mice that lack the GR encoded by the Nr3c1 gene specifically for dopamine receptor 1a^18^.

Consistent with our previous study^25^, we detected recombination of the *GR* floxed allele only in VTA, suggesting that the D-KO mouse used in the present study is a model animal in which GR is selectively deleted in VTA. On the other hand, it is still unclear from this study whether GR deficiency in VTA affected the neural activity of dopaminergic neurons, as we did not evaluate dopamine concentrations. This is a limitation and further studies are required to clarify how glucocorticoids affect the dopaminergic neuronal system in the VTA.

In conclusion, glucocorticoid signaling in dopaminergic neurons of the VTA potentiates the rewarding value of a HFD under restricted caloric conditions.

## Methods

### Mice

All animal procedures were approved by the Animal Care and Use Committee of Nagoya University Graduate School of Medicine and performed in accordance with the institutional guidelines that conform to the National Institutes of Health animal care guidelines. The study was carried out in compliance with the ARRIVE guidelines. Mice were maintained on a 12-h light (9:00–21:00) /12-h dark cycle (21:00–9:00) in a temperature-controlled barrier facility, with free access to water and food. Age-matched littermates were used for all experiments.

### Mice with DAT-specific and GPR88-specific deletion of GR

*GR*^*loxP/loxP*^ mice previously generated by Dr. Günther Schütz^26^ were provided by EMMA(ID 02,124). *DAT-Cre* transgenic mice, in which the locus of Cre-recombinase is in the 3’ untranslated region of *DAT* gene^27^ (hereafter termed *DAT-Cre* mice), were provided by Jackson Laboratory (JAX stock #006,660). *GPR88-Cre* transgenic (RRID:IMSR_RBRC10287) mice (hereafter termed *GPR88-Cre* mice) express functional Cre-recombinase mainly in medium spiny neurons and a small population of parvalbumin-positive interneurons in the caudate-putamen and NAc^14,15^. *ROSA26* Cre-reporter knock-in C57BL/6 N mice (RBRC 04,874), which exhibit green emission before and red after Cre-mediated recombination^16^ (hereafter termed *R26GRR*), were provided by RIKEN BRC through the National Bio-Resource Project of the Ministry of Education, Culture, Sports, Science and Technology, Japan. Primer sequences used for genotyping are shown as Table 1. DNA was extracted from the tail of each experimental mouse at the age of 10 days to check for the occurrence of a spurious germline deletion using the primers of *GR*Δ/Δ and *GAPDH* (for an internal control). All *GR*^*loxP/loxP*^ mice, *DAT-Cre* mice, *GPR88-Cre* mice, and *R26GRR* mice were backcrossed more than 10 generations onto a C57BL/6 background.

**Table 1:** Primers used in the present study.

Gene	Forward primer (5’ → 3’)	Reverse primer (5’ → 3’)
*GR*^*loxP/loxP*^	GGCATGCACATTACTGGCCTTCT	CCTTCTCATTCCATGTCAGCATGT
*DAT-Cre*	TGGCTGTTGGTGTAAAGTGG	GGACAGGGACATGGTTGACT [to detect wild-type (WT) gene]
		CCAAAAGACGGCAATATGGT (to detect transgene)
*GPR88-Cre*	ACCTGATGGACATGTTCAGGGATCG	TCCGGTTATTCAACTTGCACCATGC
*R26GRR*	AAAGTCGCTCTGAGTTGTTAT	CTTGTACAGCTCGTCCATGCCGAG
*GR*Δ/Δ	GGCATGCACATTACTGGCCTTCT	GTGTAGCAGCCAGCTTACAGGA
*TH*	TTGAAGGAACGGACTGGCTT	GAAACACACGGAAGGCCAGA
*ΔFosB*	AGGCAGAGCTGGAGTCGGAGAT	GCCGAGGACTTGAACTTCACTCG
*D1R*	GTAGCCATTATGATCGTCAC	GATCACAGACAGTGTCTTCAG
*GAPDH (control)*	AACGACCCCTTCATTGAC	TCCACGACATACTCAGCAC

### Isolating DNA from tissues for detection of recombination of floxed alleles

Tissues of mice at the age of 8 weeks were digested with 50 mM NaOH for 10 min at 95 °C, and 1 M Tris–HCl (pH 8.0) was added to the digestion. Samples were centrifuged for 10 min at 12,000 g and supernatants were transferred to a fresh tube. DNA was subjected to genotyping analyses by PCR with KOD FX DNA polymerase (Toyobo, Osaka, Japan) and the oligonucleotide primers. The PCR was performed with SimpliAmp Thermal Cycler (Thermo Fisher Scientific, MA, USA). The condition was 5 min at 95℃ followed by 30 cycles at 95℃ for 30 s, 56℃ for 20 s and 72℃ for 60 s with a 7 min final extension. After mixed with 6 × loading dye (Toyobo), the amplified products were electrophoresed with 1.5% agarose gel (Thermo Fisher Scientific) containing ethidium bromide (Merck Millipore, Darmstadt, Germany). DNA bands were visualized under ultraviolet light using Quantity-One software (Bio-Rad, CA, USA).

### Body composition and measurement of BW

At weaning (3 weeks old), mice were placed on diets of either standard chow CE-2 (chow); calories provided by protein (24.9%), fat (4.6%), and carbohydrate (70.5%) (CLEA, Tokyo, Japan) or a custom HFD (58Y1); calories provided by protein (18.3%), fat (60.9%), and carbohydrate (20.1%) (Test diet). The measurement of BW in HFD-fed mice was performed until the age of 16 weeks, when mice were sacrificed. Epididymal fat pat weight, brown adipose tissue weight, and blood glucose levels were measured at the beginning of the light cycle (between 09:00 and 10:00), when mice were in the fed state.

### Measurement of serum corticosterone levels

Blood was collected via submandibular bleeding from mice. Serum was separated by centrifugation at 6000 rpm for 10 min and corticosterone levels were measured by enzyme-linked immunosorbent assay (AssayPro, MO, USA).

### Brain collection for immunohistochemistry

Mice were deeply anesthetized and transcardially perfused with a cold fixative containing 4% paraformaldehyde (PFA) in phosphate-buffered saline (PBS) pH 7.4, between 09:00 and 10:00 in the fed state. After fixation, brains were removed and immersed in the same fixative for 2 h at 4 °C. The brains were kept in PBS containing 10%-20% sucrose at 4 °C for cryoprotection. They were embedded in Tissue-Tek O.C.T. compound (Sakura Finetek, Tokyo, Japan) and stored at -80 °C until sectioning. Brains were cut into 20-μm sections on a cryostat at -20 °C, thawed and mounted on Superfrost Plus microscope slides (Matsunami, Tokyo, Japan), and stored at -80 °C until immunohistochemistry was performed as described previously^28^.

### Immunohistochemistry

The frozen sections were washed with PBS, 0.3% Triton X-100 in PBS (15 min) and 50 mM glycine (15 min) followed by blocking with a mixture of 3% bovine serum albumin (Wako, Osaka, Japan) in PBS for 1 h at room temperature. Next, the sections were incubated with anti-DAT antibody (1:500; Merck Millipore)　or anti-GR (1:500; Santa Cruz Biotechnology, CA, USA) overnight at 4 °C. The sections were then treated with Alexa Fluor 488-conjugated anti-rat IgG secondary antibody (1:1,000; Thermo Fisher Scientific), Alexa Fluor 594-conjugated anti-rabbit IgG secondary antibody (1:1,000; Thermo Fisher Scientific) or Alexa Fluor 647-conjugated anti-rabbit IgG secondary antibody (1:1,000; Thermo Fisher Scientific), and DAPI (1:1000; Dojindo, Kumamoto, Japan) for 1 h at room temperature. After washing in 1 × PBS, sections were placed on slides, air dried, and cover slipped with Vectashield (Vector Labs, CA, USA). All fluorescently stained sections were examined with a confocal laser microscope (TiEA1R; Nikon Instech, Tokyo, Japan) and viewed using NIS-Elements software (Nikon Instech).

### Caloric restriction and conditioned place preference test

Animals used in this experiment consisted of 14–16 week-old KO and WT mice fed with chow. As shown in Supplementary Figure 1, mice underwent a 19-day protocol consisting of caloric restriction, limited access to HFD, and CPP. All the experiments in this session were performed using caloric-restricted mice that were provided ad libitum access to chow or HFD for a continuous 4-h period daily between 15:00–20:30, and this caloric restriction was maintained throughout the protocol. From day 4 to 8, mice were fed HFD for 2 h followed by chow for 2 h to avoid neophobia and to induce addiction to HFD. The conditioned place preference test was performed according to previously reported methods^17,29^. The apparatus consisted of 2 compartments: a white Plexiglas box and a black Plexiglas box (16 × 16 × 7 cm). To enable a mouse to distinguish the boxes, the floor of the white box was covered with uneven Plexiglas, and that of the black box, with smooth black Plexiglas. The place conditioning schedule consisted of 3 phases: preconditioning, conditioning, and testing. In the preconditioning phase, each mouse was allowed to move freely between the boxes for 20 min once a day during 2 days (day 7 and 8). On day 8, the time spent in each of the boxes was measured using a MED-PC IV (Neuroscience Idea, Osaka, Japan). Conditioning was counterbalanced between compartments so as to ensure the procedure was unbiased. Conditioning sessions were performed on days 9 to 18. On even days, each mouse was confined to 1 conditioning compartment in the presence of HFD pellets for 30 min. On odd days, each mouse was confined to the other conditioning compartment in the presence of a chow pellet for 30 min. After conditioning, each mouse was fed chow for 3.5 h in their home cage every day. On day 19, the testing phase was performed similarly to the preconditioning phase, with free access to both compartments for 20 min, and the time spent in each of the boxes was measured. Conditioned place preference was calculated for each mouse as the difference in time spent in the HFD compartment between the preconditioning and testing sessions. The control mice underwent the same protocol with chow instead of HFD throughout the protocol.

### Analysis of mRNA expression levels

The VTA and NAc of WT and D-KO mice were dissected from mice fed a chow or mice with caloric restriction. Caloric restriction in this session were performed based on the same protocol as CPP, by which mice were provided ad libitum access to chow for a continuous 4-h period daily between 16:00–20:00 for 19 days. Before dissection, mice were deeply anesthetized using 2.0% isoflurane with an animal anesthetizer device (MK-AT210; Muromachi Kikai, Tokyo, Japan). Immediately after removing, the brain tissue was stored at − 80 °C until analysis. Total RNA was extracted from samples using TRIzol (Thermo Fisher Scientific) and the RNeasy kit (QIAGEN, Hilden, Germany). cDNA was synthesized from 100 ng total RNA using the ReverTra Ace qPCR RT Kit (Toyobo). Quantitative real-time polymerase chain reaction (qRT-PCR) was performed using Brilliant III Ultra-Fast SYBR Green QPCR Master Mix (Agilent Technologies, Santa Clara, CA), and samples were run using the CFX Connect Real-Time PCR Detection System (Bio-Rad). The relative mRNA levels of *TH (tyrosine hydroxylase)* in VTA, *Fosb (FBJ osteosarcoma oncogene B)* and *Drd1 (dopamine receptor D1)* in NAc were assessed by qRT-PCR using *Gapdh* as an internal control. Relative mRNA expression levels were calculated using the comparative Ct method as described previously^28^. Primer sequences are listed in Table 1.

### Novel object recognition test

The novel object recognition test (NORT) was performed according to previously reported methods^30,31^. The experimental apparatus consisted of a Plexiglas open-field box (30 × 30 × 35 high cm), the floor of which was covered in sawdust. The apparatus was located in a sound-attenuated room and was illuminated at an intensity of 15 Lux. Shown in Supplementary Figure 4, the NORT procedure consisted of 3 sessions: habituation, training, and retention sessions. Each mouse was individually habituated to the box, with 10 min of exploration in the absence of objects each day for 3 consecutive days (habituation session, days 1–3). During the training session, 2 novel objects were symmetrically fixed to the floor of the box, 8 cm from the walls, and each animal was allowed to explore in the box for 10 min (day 4). The objects were constructed from a golf ball, wooden column, and wall socket, which were different in shape and color but similar in size. The animals were considered to be exploring the object when the head of the animal was facing the object or the animal was touching or sniffing the object. The time spent exploring each object was recorded. During the retention sessions, the animals were placed back into the same box 24 h (day 5) after the training session, in which one of the familiar objects used during training was replaced by a novel object. The animals were then allowed to explore freely for 5 min and the time spent exploring each object was recorded. Throughout the experiments, the objects were used in a counterbalanced manner in terms of their physical complexity and emotional neutrality. A preference index, a ratio of the amount of time spent exploring any 1 of the 2 objects (training session) or the novel object (retention session) over the total time spent exploring both objects, was used to measure cognitive function.

### Statistical analysis

Statistical significance of the differences between groups was analyzed by either unpaired t test, two-way factorial ANOVA or two-way ANOVA with repeated measures followed by Bonferroni’s test. Results are expressed as mean ± SEM and differences were considered statistically significant at *p* < 0.05.

## Supplementary Information

Supplementary Information.

## Data Availability

The datasets generated and/or analyzed during the current study are available from the corresponding author on reasonable request.
