# Supplementary material for: Glucocorticoid receptor signaling in ventral tegmental area neurons increases the rewarding value of a high-fat diet in mice
Source: Sci Rep. 2021 Jun 18;11:12873. doi: 10.1038/s41598-021-92386-7 (PMC8213822; doi:10.1038/s41598-021-92386-7)
Supplement: Supplementary file 1 — Supplementary Information. [file 41598_2021_92386_MOESM1_ESM.pdf]

Figure S1

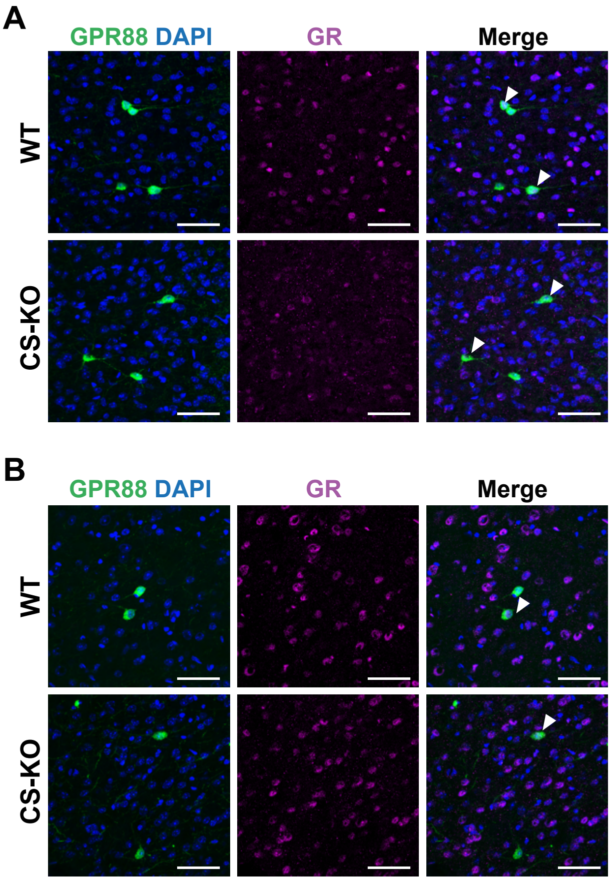

Supplementary Figure 1. GR expression in the tdsRed-positive cells in mPfc and Ofc in WT and CS-KO mice.

Immunostaining of GR in the mPfc (A) and Ofc (B) of mice. Representative photographs show the staining of GR (red) and DAPI (blue) in mPfc and Ofc in GPR88-GR+/+ R26GRR mice (WT) and GPR88-GR-/- R26GRR mice (CS-KO). mPfc, medial prefrontal cortex; Ofc, orbitofrontal cortex. Scale bar: 50 μm.

Figure S2

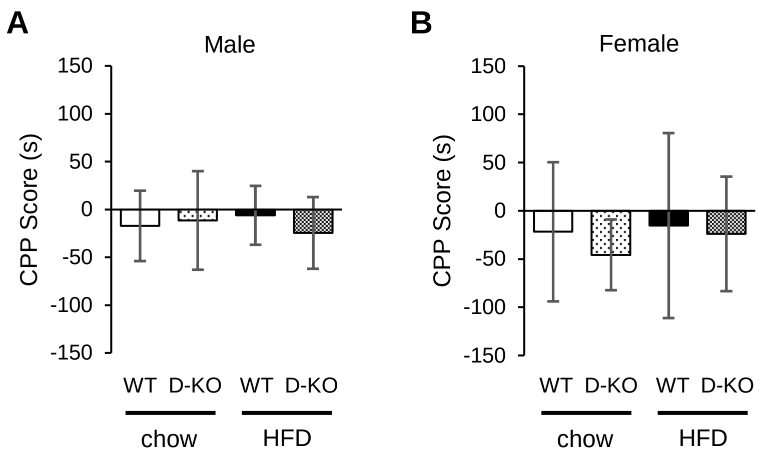

Supplementary Figure 2. Preference for HFD was not observed in the CPP test under non-stressed condition in WT and D-KO mice.

Results of the CPP test without caloric restriction in WT and D-KO mice. Either genotype exhibited no significant preference for the HFD-paired compartment, either males or females (A: male n=5-8, B: female n=5-6 for each group). All values are mean ± SEM. Statistical analyses were performed using two-way factorial ANOVA followed by Bonferroni post hoc test.

Supplementary Figure 3. Protocol for caloric restriction and conditioned place preference test (CPP).

Day 1-6

preparation

From day 1 through 19, each mouse was underwent caloric restriction.
- ad libitum access to chow or HFD for continuous 4 hours as below:
day 1-3: chow 4h
day 4-8: HFD 2h and chow 2h
day 9-18:
<even days> HFD 30min (in the compartment) and chow 3.5h (home cage)
<odd days> chow 30min (in the compartment) and chow 3.5h (home cage)
day 19: chow 4h

Day 7-8

preconditioning

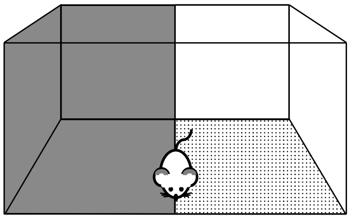

Each mouse was allowed to move freely between two boxes for 20min.
On day 8, the time spent in each of the boxes was measured.

Day 9-18

conditioning

<Even days>

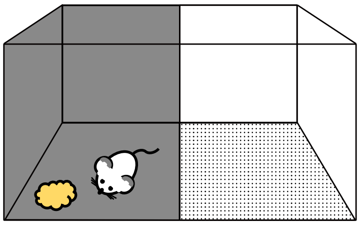

Each mouse was confined to one compartment in the presence of HFD pellet for 30 min.

<Odd days>

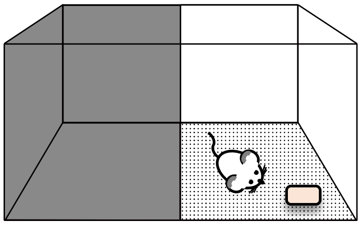

Each mouse was confined to the other compartment in the presence of chow pellet for 30 min.

After the conditioning, each mice was fed with chow for 3.5 hours in their home cage every day.

Day 19

testing

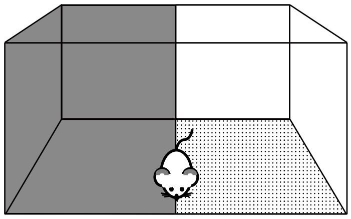

Each mouse was allowed to move freely between two boxes for 20min.
The time spent in each of the boxes was measured.
Conditioned place preference was calculated for each mouse as the difference in time spent in the HFD compartment between preconditioning and testing session.

Supplementary Figure 4. Protocol for novel object recognition test (NORT).

Day 1-3

habituation

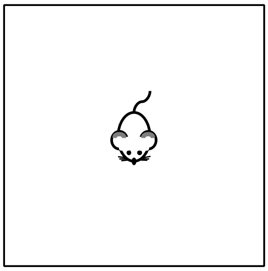

Each mouse was allowed to explore in the box for 10min,
in the absence of objects.

Day 4

training

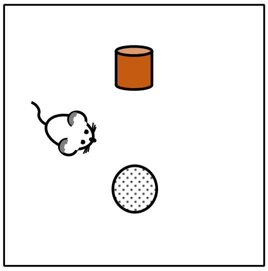

Two novel objects（a golf ball and a wooden column）were
symmetrically fixed to the floor of the box.
Each mouse was allowed to explore in the box for 10 min.
The time spent exploring each object was recorded.

Day 5

retention

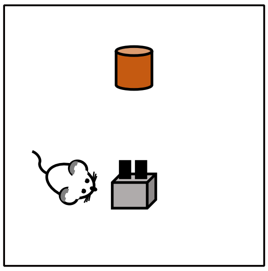

During the retention session, one of the familiar objects
was replaced by a novel object (a wall socket).
The mouse was allowed to explore in the box for 5 min.
The time spent exploring each object was recorded.

Supplementary Figure 5. Whole gels of Figure 1A and 1B.
Whole gels of Figure 1A and B. Manuscript figure is indicated above the gels.

Figure 1A

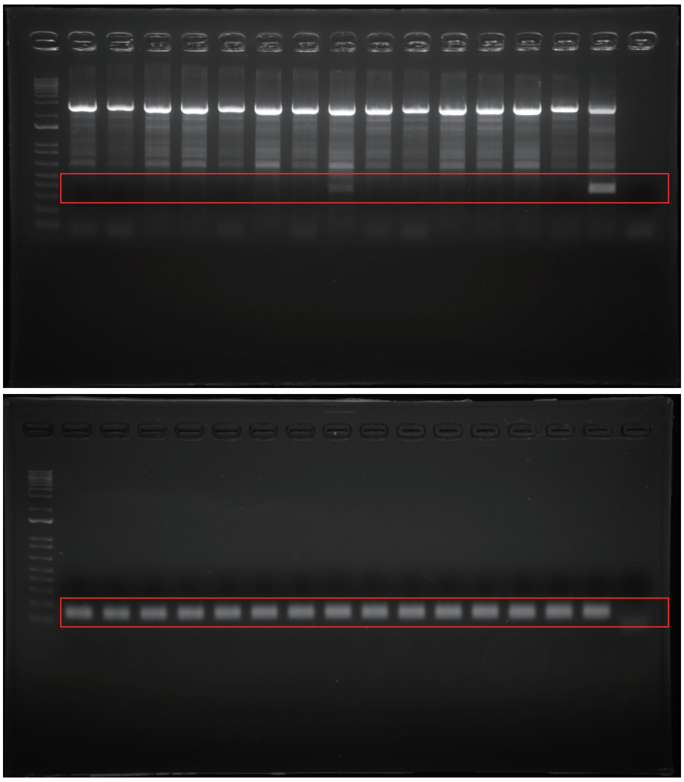

GR Δ allele

GAPDH

Figure 1B

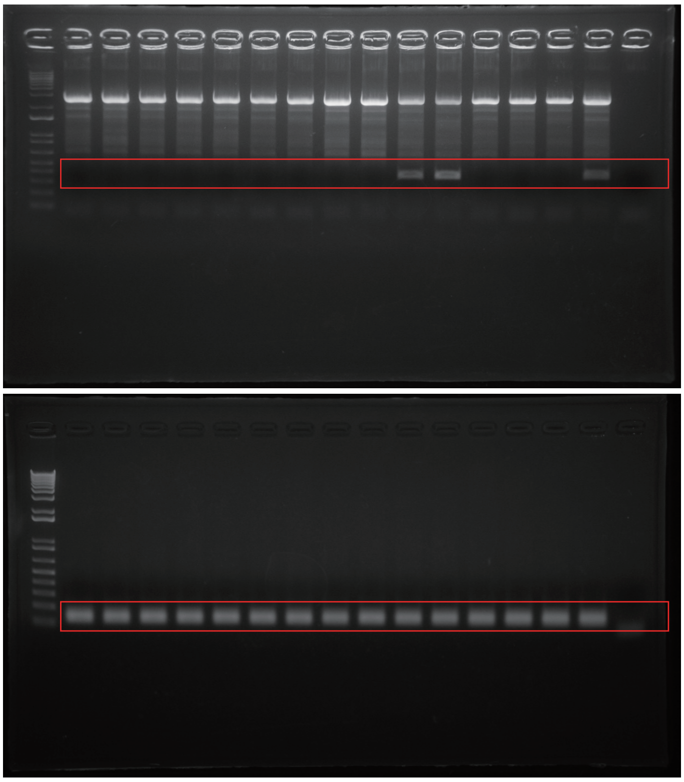

GR Δ allele

GAPDH
